# Apnoeic Oxygenation Using High-Flow Oxygen: Effects on Partial Pressure of Carbon Dioxide in Rigid Bronchoscopy

**DOI:** 10.3390/jcm14228064

**Published:** 2025-11-14

**Authors:** Bon-Sung Koo, Yang-Hoon Chung, Misoon Lee, Sung-Hwan Cho, Jaewoong Jung

**Affiliations:** Department of Anaesthesiology and Pain Medicine, Soonchunhyang University Bucheon Hospital, Soonchunhyang University College of Medicine, Bucheon-si 14584, Republic of Korea; kbs0803@schmc.ac.kr (B.-S.K.); drcyh79@schmc.ac.kr (Y.-H.C.); misoonlee@schmc.ac.kr (M.L.); singaring@schmc.ac.kr (S.-H.C.)

**Keywords:** apnoea, bronchoscopy, insufflation, hypercapnia, acidosis, respiratory

## Abstract

**Background/Objectives**: Rigid bronchoscopy poses safety challenges due to airway leakage. Although apnoeic oxygenation is a potential strategy, concerns over carbon dioxide (CO_2_) retention have limited its adoption. The introduction of high-flow nasal cannula (HFNC) has renewed interest by potentially mitigating CO_2_ accumulation during prolonged apnoea. This study investigated changes in the arterial partial pressure of CO_2_ (PaCO_2_) during apnoeic oxygenation using Optiflow™. **Methods**: We retrospectively analysed patients undergoing rigid bronchoscopy with HFNC (70 L·min^−1^) from 2020 to 2022. The apnoeic period was defined from the onset of apnoeic oxygenation to ventilation resumption. Arterial blood gas levels and complications, including arrhythmia and desaturation, were evaluated. Regression analysis was used to evaluate changes over time. **Results**: Apnoeic oxygenation was performed in 10 male patients (mean age 65 ± 14 years; body mass index 24.75 ± 4.18 kg·m^−2^). The mean duration of apnoea was 33.7 ± 13.7 min, with PaCO_2_ rising linearly at 1.50 mmHg/min. No interventions were required to maintain SpO_2_ above 91% for all patients. Except for one case of atrial fibrillation that occurred during emergence rather than the apnoeic period, no significant complications were observed. **Conclusions**: The observed increase in PaCO_2_ was lower than in previously reported studies using HFNC via the nares, suggesting that direct delivery of oxygen to the distal airway via bronchoscopy may enhance CO_2_ clearance through more effective washout. Apnoeic oxygenation with HFNC could potentially overcome airway leakage for selected patients, but vigilant monitoring remains essential throughout the apnoeic period. Further research is warranted to enhance patient safety.

## 1. Introduction

Several ventilatory strategies, including apnoeic oxygenation, controlled ventilation, and jet ventilation, are recommended for rigid bronchoscopy. However, the use of apnoeic oxygenation is limited due to concerns regarding carbon dioxide (CO_2_) accumulation, leading to the risk of respiratory acidosis and cardiac arrhythmia [1]. Recently, the use of high flow nasal cannula (HFNC) has gained popularity, as it facilitates oxygenation while attenuating the increase in CO_2_ levels. Apnoeic oxygenation using HFNC has been safely and effectively applied during airway surgeries, such as laryngomicrosurgery [2].

The physiological basis of apnoeic oxygenation relies on a subatmospheric pressure gradient, generated as oxygen (O_2_) absorbed from the alveoli into the bloodstream, which drives the movement of O_2_ from the upper airway to the alveoli [3]. Increasing the flow rate of O_2_ allows O_2_ to reach more distal airways and generates a positive airway pressure that is proportional to the flow, thereby prolonging the duration of apnoea without desaturation (DAWD). Furthermore, O_2_ delivery to more distal sites, such as the trachea or bronchi, may further extend DAWD compared to delivery to the upper airway [4].

The bronchial bronchoscope, which is thinner and longer than the tracheal bronchoscope, results in increased leakage, complicating ventilation and increasing susceptibility to desaturation during the procedures. When using HFNC, the bronchoscope might serve as a conduit to deliver O_2_ directly to the alveoli, potentially enhancing oxygenation and CO_2_ clearance; however, there is little clinical evidence supporting this mechanism. Beyond this theoretical possibility, HFNC could provide an uninterrupted and stable procedural field, minimising motion artifacts and optimizing visualization.

In this study, we investigated changes in the partial pressure of CO_2_ (PaCO_2_) in patients undergoing apnoeic oxygenation during rigid bronchoscopy. Additionally, we assessed changes in the partial pressure of O_2_ (PaO_2_), pH, and the incidence of potential complications such as cardiac arrhythmia.

## 2. Materials and Methods

This retrospective study was conducted at a tertiary regional hospital in South Korea. The study protocol was approved by the Institutional Review Board (IRB) of Soonchunhyang University Bucheon hospital (IRB No. 2023-12-001; Chairperson Dr. Seong Kyu Park) on 22 December 2023, and informed consent was waived due to the retrospective design.

### 2.1. Participants and Data Collection

We included patients aged 18 years or older who underwent rigid bronchoscopy under general anaesthesia between May 2020 and March 2022. Only those who received apnoeic oxygenation using Optiflow™ (Fisher & Paykel Healthcare, Auckland, New Zealand) at a flow rate of 70 L·min^−1^ during the procedure were eligible. Optiflow™, a commercially available HFNC device, was set to deliver oxygen only for rapid use in the operating room, with flow rates of up to 70 L·min^−1^. Patients without an arterial line for arterial blood gas analysis (aBGA) were excluded from the study. Further exclusion criteria encompassed pulmonary diseases influencing oxygenation, apart from the primary lesion requiring bronchoscopy, and cardiac diseases, such as arrhythmias, ischemic heart disease, or heart failure, owing to potential complications during apnoeic oxygenation.

The primary outcome was the change in PaCO_2_ during the apnoeic period; secondary outcomes included changes in PaO_2_ and pH. We collected the results of aBGA from electronic medical records to evaluate serial changes in these parameters (Shown in Table S1). Additional data, including demographics, procedural details, and vital signs, were also extracted from the medical records and VitalDB database. The VitalDB program is a platform that synchronously records intraoperative patient data from various monitoring devices, providing a comprehensive dataset [5].

Apnoea onset was defined as the absence of end-tidal CO_2_ (EtCO_2_) with initiation of apnoeic oxygenation using HFNC following bronchoscope insertion. The end of apnoea was defined as resumption of ventilation, confirmed by the reappearance of EtCO_2_ following completion of the procedure. The apnoeic period was the interval between these points.

### 2.2. Anaesthesia Protocol

All patients were managed according to a standardised institutional protocol during rigid bronchoscopy. Upon arrival in the operating room, standard American Society of Anaesthesiologists (ASA) monitoring was applied, including non-invasive blood pressure, electrocardiography (EKG), and peripheral oxygen saturation (SpO_2_). Preoxygenation was performed at the discretion of the attending anaesthesiologist using either a facemask (with 100% oxygen at 6 L·min^−1^ until an end-tidal oxygen concentration exceeding 90%) or HFNC (at 30 L·min^−1^ for 3 min). Anaesthesia was induced with total intravenous anaesthesia using propofol and remifentanil by target-controlled infusion at effect-site concentrations of 4–5 ng·mL^−1^ and 1–2 ng·mL^−1^, respectively. Neuromuscular blockade was achieved with rocuronium (0.6–0.8 mg·kg^−1^), and endotracheal intubation was performed. The pulmonologist subsequently extubated the endotracheal tube and inserted a rigid bronchoscope. The bronchial bronchoscope inevitably creates a leak around the airway. The absence of leakage, particularly when high-flow oxygen is applied, carries a substantial risk of severe complications associated with barotrauma or volutrauma. Therefore, to ensure patient safety, the anaesthesiologist connected the ventilator circuit to the bronchoscope port and performed manual ventilation to confirm leakage. After confirming leakage via manual ventilation (indicated by collapse or incomplete inflation of breathing bag, or by an audible hissing), the HFNC circuit was connected to the bronchoscope port at a rate of 70 L·min^−1^. An aBGA sample was obtained at the initiation of HFNC, followed by serial samples collected at 5-min intervals. Throughout the procedure, vital signs, especially SpO_2_ and EKG, and leakage were closely monitored. Apnoea was immediately terminated if SpO_2_ dropped below 90%, requiring additional intervention, or if other complications, such as arrhythmia or significant EKG changes, were observed. Upon completion of the procedure, the rigid bronchoscope was removed, and either the endotracheal tube or a supraglottic airway device was re-inserted to resume ventilation. Neuromuscular blockade was reversed with 200 mg of sugammadex, and patients were awakened and transferred to the post-anaesthesia care unit, where the final aBGA was performed.

### 2.3. Statistical Analysis

All analyses were performed using SPSS version 23.0 (IBM Corp, Armonk, NY, USA). The normality of continuous variables was assessed using the Shapiro–Wilk test. Normally distributed variables are presented as mean ± standard deviation, and non-normally distributed variables are given as median [interquartile range]. Categorical variables are expressed as number (percentage). Linear mixed-effect modelling (LMM) was used to account for repeated measures and within-patient variability. To address the reduced sample size and consequent data asymmetry at longer procedure times, stratified analyses were conducted using a cutoff of 35 min for apnoea duration. Because true baseline (0 min) PaCO_2_ data were unavailable, the PaCO_2_ measured at 5 min after apnoea onset was used as the initial value for comparison. The changes in PaCO_2_ (ΔPaCO_2_ = Final PaCO_2_ − PaCO_2_ at 5 min) and the PaCO_2_ accumulation rate (ΔPaCO_2_ divided by apnoea duration) were compared between the two groups. Between-group comparisons were conducted using the independent t-test or Mann–Whitney U test, as appropriate based on data distribution. For analysis of pH, linear regression was conducted using natural log-transformed time (ln(time)) as the independent variable. A *p*-value < 0.05 was considered statistically significant. Missing values were not imputed.

## 3. Results

Twelve patients underwent rigid bronchoscopy with apnoeic oxygenation using HFNC. Two were excluded due to a lack of arterial line placement. Consequently, data from 10 patients with reliable aBGA were included in the final study cohort.

All patients were male, with a mean age of 65 ± 14 years (range: 42–82 years) and mean body mass index (BMI) of 24.75 ± 4.18 kg·m^−2^ (range: 16.93–28.79 kg·m^−2^). ASA physical status classification and smoking history are summarized in Table 1.

The mean total apnoea time was 37.7 ± 13.7 min (range: 19–67.5 min). Throughout the apnoeic period, SpO_2_ remained above 91% in all patients except Case 3, who experienced a transient drop of 82% attributed to tumour-related bronchial obstruction. After resolution of the obstruction via cryotherapy, SpO_2_ recovered and remained above 92% for the remainder of the procedure. No interventions were required during apnoeic oxygenation. Detailed results are presented in Table 2.

**Figure 1:**
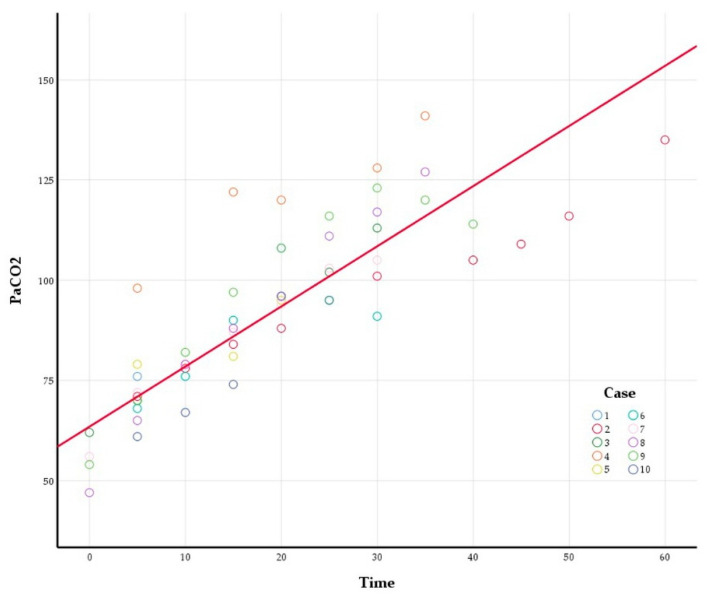
Time-dependent trajectory of PaCO_2_ during the apnoeic period. Dots represent individual measurements for each subject; the red line represents the fitted trend from the linear mixed-effects model; PaCO_2_ = 63.45 + 1.50 × time (min).

All individual PaCO_2_ measurements are plotted as data points in Figure 1. A significant upward trend in PaCO_2_ was observed over time (*p* < 0.001). Specifically, PaCO_2_ was estimated to increase by 1.50 mmHg per min (95% confidence interval, 1.25–1.76 mmHg). Furthermore, a significant positive correlation was observed among repeated PaCO_2_ measurement within individual patients (AR(1) rho = 0.691, *p* < 0.001).

Patients were stratified into two subgroups based on an apnoea duration of 35 min (≤35 min group, >35 min group). Changes in PaCO_2_ accumulation rate were compared between groups (Table 3). There was no statistically significant difference in the PaCO_2_ accumulation rates between two groups (*p* = 0.596).

**Figure 2:**
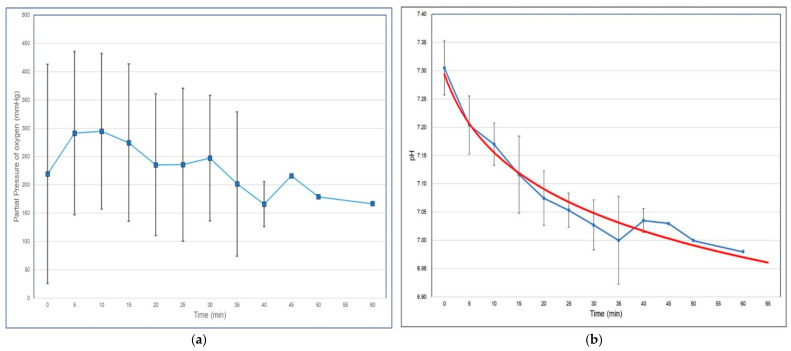
Serial changes during the apnoeic period. (**a**) Time course of partial pressure of oxygen (PaO_2_); (**b**) Time course and logarithmic regression of pH. The red curve shows the logarithmic relationship between pH and time: pH = 7.499 − 0.127 × ln (time); R^2^ = 0.963, *p* < 0.001.

Figure 2a displays the mean and standard deviation of PaO_2_ at each measured time interval: LMM analysis showed no statistically significant change in PaO_2_ over time (*p* = 0.691).

Figure 2b illustrates the changes in pH over time. For pH, regression analysis using log-transformed time showed a strong inverse association.

No adverse events attributable to apnoeic oxygenation were observed throughout the procedures. One patient (Case 5) developed atrial fibrillation during emergence from anaesthesia; all patients recovered uneventfully and were transferred to the post-anaesthesia care unit (PACU) without additional complications. Acute respiratory acidosis induced by prolonged apnoea was promptly corrected and resolved to normal limits in all patients, as confirmed by follow-up aBGA measurement in the PACU.

## 4. Discussion

In this retrospective study, we investigated serial changes in PaCO_2_ and PaO_2_ during apnoeic oxygenation with high-flow O_2_ delivered directly into the distal airway via a rigid bronchoscope. We found that PaCO_2_ increased linearly at a rate of 1.50 mmHg per minute, while PaO_2_ did not exhibit a significant decline during prolonged apnoeic period. Notably, no intervention was needed, and all patients recovered without major complications, except for a single case of atrial fibrillation during emergence, not during an increase in or at a peak level of CO_2_. It remains unclear whether this arrhythmia was associated with apnoeic oxygenation or with patient-related factors such as advanced age and comorbidities. Nevertheless, careful attention remains warranted when employing apnoeic oxygenation.

During apnoeic oxygenation, the subatmospheric pressure that moves O_2_ into the alveoli also facilitates the transfer of accumulated arterial CO_2_ to the alveoli [3]. High flow rates produce turbulent flow that reaches the distal airways, enhancing CO_2_ clearance and thereby reducing the rate of CO_2_ accumulation [4]. Patel et al. [6] reported that high-flow O_2_ insufflation could decrease the rate of increase in CO_2_ from 2.63 to 3.38 mmHg·min^−1^, as seen in classical apnoeic oxygenation, to 1.13 mmHg·min^−1^. Similarly, Gustafsson et al. [7] observed a rate of PaCO_2_ increase of 1.80 mmHg·min^−1^ when using HFNC.

As the flow rate increases, DAWD during apnoeic oxygenation can be extended. Importantly, beyond simply delivering high flow, the location of O_2_ administration plays a crucial role in determining DAWD. While Optiflow™ delivers high-flow O_2_ via the nostril, our study shows that O_2_ can also be administered directly to the distal airway, such as the trachea or bronchi. Supplying O_2_ to more distal airways is thought to have a greater impact on DAWD [4]. In practice, our study achieved a mean apnoea duration of 37.7 min without requiring additional intervention for SpO_2_ desaturation. Direct delivery of O_2_ to the distal airway likely promotes greater washout and turbulence, further enhancing CO_2_ clearance. The rate of CO_2_ increase was 1.37 mmHg/min in our study, lower than the 1.80 mmHg min^−1^ reported by Gustafsson et al. [7] but higher than the 1.13 mmHg min^−1^ reported by Patel et al. [6]. Notably, Patel et al. used both EtCO_2_ and PaCO_2_ measurements; because the difference between EtCO_2_ and PaCO_2_ widens with longer apnoea times [8], EtCO_2_ tends to underestimate the PaCO_2_, possibly explaining their lower calculated values compared to ours.

In other studies of apnoeic oxygenation during rigid bronchoscopy, an animal experiment reported a PaCO_2_ increase of 4 mmHg min^−1^ with 15 L·min^−1^ O_2_ [9]. In a study that used 70 L·min^−1^, similar to our protocol, with aBGA performed every 10 min, PaCO_2_ increased to 76 mmHg after an average of 23.9 min of apnoea [10]. When comparing results reported with EtCO_2_, a group ventilated with 6 L·min^−1^ during apnoea showed greater CO_2_ accumulation (55.2 mmHg) than a high flow of 50 L·min^−1^, with maximum CO_2_ of 51.8 mmHg [11]. There are also reports that applying HFNC while maintaining spontaneous breathing during rigid bronchoscopy allows both efficient oxygenation and effective CO_2_ removal [12].

Based on the above findings, direct administration of high-flow O_2_ into the distal airway may enable apnoeic ventilation, in which both oxygenation and CO_2_ clearance are achieved, beyond the concept of apnoeic oxygenation. However, this approach is not without risks: it increases the risk of barotrauma and volutrauma. Animal studies have shown that inflating the cuff of an endotracheal tube during 15 L·min^−1^ O_2_ delivery can cause a dangerous rise in airway pressure, whereas deflating the cuff avoids this hazard [9]. In our study, administration of 70 L·min^−1^ could have resulted in catastrophic outcomes during even a brief closed-circuit interval. To mitigate this risk, one anaesthesiologist continuously monitored for leakage by listening for a distinct hissing sound and ensuring that the bag of a bag-valve-mask system did not inflate throughout the entire procedure; the other anaesthesiologist monitored vital signs.

Our study had several limitations. First, the small sample size (*n* = 10) limits the statistical power and generalisability of the results. Although previous studies have demonstrated the efficacy of high-flow O_2_ during rigid bronchoscopy, larger studies are needed to confirm our findings. Second, as a retrospective study, selection and interpretation bias may have occurred. All 10 cases were male patients in our study; given potential differences in lung function and metabolism between the sexes, further studies including female patients are needed. Third, direct evidence regarding patient safety during apnoea with HFNC remains limited. Future studies should enhance patient safety by incorporating direct monitoring of critical parameters, such as airway pressure and lung volumes. Furthermore, establishing specific safety thresholds for PaCO_2_ and pH and investigating the clinical implications of hypercapnia resulting from prolonged apnoea is crucial. In addition, alternative monitoring for CO_2_, such as transcutaneous CO_2_ monitoring, may be a helpful consideration [13,14]. Lastly, rather than using a fixed high flow rate of 70 L·min^−1^, future research should identify the optimal flow rate that maximises oxygenation and CO_2_ clearance while minimising the risks of barotrauma and volutrauma.

## 5. Conclusions

In conclusion, apnoeic oxygenation at a high flow rate of 70 L·min^−1^ during rigid bronchoscopy resulted in a linear increase in PaCO_2_ of 1.50 mmHg·min^−1^. For patients in whom severe leakage precludes the use of intermittent manual ventilation or controlled ventilation, an adequate flow of O_2_ can allow for the maintenance of oxygenation while attenuating CO_2_ accumulation. This study demonstrates the potential applicability of apnoeic oxygenation during rigid bronchoscopy; however, further research is required to determine the optimal oxygen flow rate that minimizes complications such as barotrauma and volutrauma, enhances patient safety, and elucidates the underlying physiologic mechanisms.

## Figures and Tables

**Table 1:** Patient characteristics and procedural data for rigid bronchoscopy.

Case	Age (Years)	BMI (kg/m^2^)	ASA Physical Status	Smoking History (Non-/Smoker/Ex-)	Diagnosis	Procedure Performed
1	55	16.93	2	Ex-smoker	Non-small cell lung cancer	Endoscopic excision of bronchus lesion, Rt
2	42	28.54	2	Non-smoker	Pulmonary alveolar proteinosis	Bronchoscopic biopsy of lung, both
3	74	25.31	3	Ex-smoker	Non-small cell lung cancer	Bronchoscopic mass removal, Rt
4	76	27.30	3	Ex-smoker	Diffuse interstitial lung disease	Bronchoscopic biopsy of bronchus, both
5	82	28.79	3	Non-smoker	Non-small cell lung cancer	Endoscopic excision of bronchial lesion, Lt
6	59	26.54	1	Ex-smoker	Diffuse interstitial lung disease	Bronchoscopic biopsy of bronchus, both
7	44	27.81	1	Smoker	Solitary pulmonary nodule	Bronchoscopic biopsy of lung, both
8	73	22.32	1	Non-smoker	Solitary pulmonary nodule	Bronchoscopic biopsy of lung, Rt
9	70	25.21	3	Non-smoker	Eosinophilic bronchitis	Bronchoscopic biopsy of lung, Lt
10	77	18.48	3	Smoker	Foreign body in bronchus	Bronchoscopic bronchial foreign body removal, Rt
	65 ±14	24.75 ±4.18	3:2:5(30%:20%:50%)	4:2:4 (40%:20%:40)		

ASA, American Society of Anaesthesiologists; BMI, body mass index.

**Table 2:** Outcome of apnoeic oxygenation during rigid bronchoscopy.

Case	Duration of Apnoea (min)	Minimal SpO_2_ (%)	Maximal PaCO_2_ (mmHg)	Minimal pH	Complications
1	26.8	96	108	7.07	-
2	67.5	97	135	6.98	-
3	40.3	82	113	7.02	-
4	48.7	98	141	6.90	-
5	25.9	91	95	7.11	Atrial fibrillation
6	33.6	97	96	7.06	-
7	37.5	98	105	7.04	-
8	34.5	100	127	7.02	-
9	43.6	91	123	6.97	-
10	19.0	99	74	7.20	-
	37.7 ± 13.7				

PaCO_2_, partial pressure of carbon dioxide.

**Table 3:** Stratified analysis based on an apnoea duration of 35 min.

	PaCO_2_ at 5 min	Final PaCO_2_	ΔPaCO_2_	PaCO_2_ Accumulation Rate
≤35 min group	69.8 ± 7.5	99.0 ± 19.8	29.2 ± 19.7	1.0 ± 0.5
>35 min group	71 (median)	121.6 ± 15.5	45.4 ± 11.3	0.9 (median)
*p*-value	0.346 ^a^	0.082	0.160 ^b^	0.596 ^a^

^a^, Mann–Whitney U test; ^b^, independent t-test; PaCO_2_, partial pressure of carbon dioxide.

## Data Availability

The data presented in this study are available on request from the corresponding author due to patient privacy concerns and ethical restrictions imposed by the institutional review board.

## Supplementary Materials

The following supporting information can be downloaded at: https://www.mdpi.com/article/10.3390/jcm14228064/s1, Table S1: Serial arterial blood gas analysis during apnoeic period.

## Abbreviations

The following abbreviations are used in this manuscript:

aBGA	Arterial blood gas analysis
ASA	American Society of Anaesthesiologists
BMI	Body mass index
CO_2_	Carbon dioxide
DAWD	Duration of apnoea without desaturation
EKG	Electrocardiography
EtCO_2_	End-tidal carbon dioxide
HFNC	High-flow nasal cannula
O_2_	Oxygen
PACU	Post-anaesthesia care unit
PaCO_2_	Partial pressure of carbon dioxide
PaO_2_	Partial pressure of oxygen
SpO_2_	Peripheral oxygen saturation
