# Supplementary material for: Apnoeic Oxygenation Using High-Flow Oxygen: Effects on Partial Pressure of Carbon Dioxide in Rigid Bronchoscopy
Source: J Clin Med. 2025 Nov 14;14(22):8064. doi: 10.3390/jcm14228064 (PMC12653440; doi:10.3390/jcm14228064)
Supplement: Supplementary file 1 [file jcm-14-08064-s001.zip › jcm-3952989-supplementary.pdf]

Table S1. Serial arterial blood gas analysis during apnoeic period.

	Apnoea onset	5 min	10 min	15 min	20 min	25 min	30 min	35 min	40 min	45 min	50 min	55 min	60 min	PACU
$PaCO_2$ (mmHg)	55 ± 6.2	73 ± 9.8	77 ± 2.2	90 ± 13.9	100 ± 9.9	104 ± 8.5	111 ± 13.0	129 ± 10.7	110 ± 6.4	109	116	-	135	42 ±4.6
PaO_2 (mmHg)	219 ± 194	291 ± 144	295 ± 138	275 ± 139	236 ± 125	236 ± 135	247 ± 111	202 ± 127	166 ±40	216	179	-	167	165 ± 23
pH	7.31 ±0.05	7.20 ± 0.05	7.17 ± 0.03	7.12 ± 0.06	7.07 ± 0.05	7.05 ± 0.03	7.03 ± 0.04	6.96 ± 0.06	7.04 ± 0.02	7.03	7.00	-	6.98	7.35 ± 0.05
HCO_3^-	27.1 ± 0.6	28.3 ± 1.4	27.9 ± 1.3	28.5 ± 1.3	28.8 ± 1.6	28.6 ± 1.8	28.7 ± 1.5	29.3 ± 3.0	29.3 ± 0.2	28.8	28.6	-	31.8	23.2 ± 1.7

$PaCO_2$, partial pressure of carbon dioxide; PACU, post-anaesthesia care unit; PaO_2, partial pressure of oxygen.
